# 3D nuclear organization and genomic instability in cancer

**DOI:** 10.1186/1753-6561-7-S2-K17

**Published:** 2013-04-04

**Authors:** Sabine Mai

**Affiliations:** 1 Manitoba Institute of Cell Biology, CancerCare Manitoba, University of Manitoba, Winnipeg, Canada

## 

To understand the genetic changes that occur at tumor initiation and during tumor progression, we focus on changes in nuclear architecture that promote the onset of genomic instability [1,2]. To determine changes in nuclear organization, we measure the 3D nuclear organization of telomeres, the ends of chromosomes. The measurements of telomeres allow one to trace the positions of chromosomes in interphase nuclei and, by using a fluorescent telomere-specific probe, all telomeres can be visualized in a single image.

During the past years, we have defined the organization of telomeres in nuclei of normal, immortalized and tumor cells. We have developed quantitative software that enables us to measure the three-dimensional (3D) organization of telomeres [3,4]. The parameters we measure include: number of telomeres, sizes of telomeres, nuclear distribution of telomeres, and the presence of telomeric aggregates. The latter are clusters of telomeres that are absent from normal cells. More recently, we started to automate the 3D image acquisition and analysis and we are able to scan 15 000 cells per hour [5].

We observed changes in the nuclear organization of telomeres as a result of conditional c-Myc deregulation dependent on a functional *myc box* II [6,7]. We found Epstein-Barr Virus-induced changes in 3D telomere organization and resulting genomic instability [8].

Encouraged by these findings, we focused on tumor initiation and progression in patient samples and observed cancer-associated 3D nuclear telomere changes in lymphoid and solid tumors [9-12]. Moreover, the use of 3D nuclear telomere profiling permitted, for the first time, the identification of patient (and tumor) subpopulations that were not detectable up to that point. For example, we blindly defined three distinct subpopulations that correlated with short-term, intermediate and long-term survival in glioblastoma [11]. Using the same 3D imaging approach, we defined subpopulations in myelodysplastic syndromes and acute myeloid leukemias [12]. Additional studies are currently ongoing.

We provided evidence that genomic instability is a result of these nuclear changes. Dynamic nuclear alterations directly result from 3D telomere aberrations [1,2,6,13-15]. These genomic changes include aneuploidy, Robertsonian fusions, breakage-bridge fusion (BBF) cycles with resulting terminal deletions and unbalanced translocations and continued rounds of BBF cycles. While these changes can be followed during cancer progression in patients, their true origin can only be examined in conditional expression studies or longitudinally in mouse models. Using conditional c-Myc deregulation, we demonstrated that changes in 3D telomere profiles precede the onset and propagation of genomic instability [6,13].

In Hodgkin’s lymphoma, in collaboration with Dr. Hans Knecht, we showed that mono-nucleated H cells become multinucleated Reed Sternberg (RS) cells through telomere dysfunction as measured by 3D nuclear profiles [10,16]. These changes coincide with localized shelterin dysfunction, aberrant centrosome duplication as well as spindle formation and significantly elevated levels of DNA damage foci [10,16]. Spectral karyotyping (SKY) and super resolution 3D imaging confirmed the dynamics and complexity of these genetic changes in which RS cells are the end-stage cells generated through multiple defects traced back to the H cells and propagated from there on [15]. Most recently, we have reported that Hodgkin’s patients with recurrent/non-recurrent disease display distinct 3D telomeric profiles [17].

Ongoing studies focus on a variety of cancers where clinicians currently lack the ability to define the risk of an individual patient to progress, the ability of early detection or of monitoring of disease progression. In conclusion, 3D telomere profiling provides a platform technology able to determine normal and aberrant nuclear organization that is a measure of genomic instability and cancer.

## Competing interests

There are no competing interests in this presentation.
